# New Sustainable Multilayered Membranes Based on ZrVTi for Hydrogen Purification

**DOI:** 10.3390/membranes12070722

**Published:** 2022-07-21

**Authors:** Stefano Fasolin, Simona Barison, Filippo Agresti, Simone Battiston, Stefania Fiameni, Jacopo Isopi, Lidia Armelao

**Affiliations:** 1 Institute of Condensed Matter Chemistry and Technologies for Energy (ICMATE), National Research Council (CNR), Corso Stati Uniti 4, 35127 Padova, Italy; stefano.fasolin@cnr.it (S.F.); filippo.agresti@cnr.it (F.A.); simone.battiston@cnr.it (S.B.); stefania.fiameni@cnr.it (S.F.); jacopo.isopi@icmate.cnr.it (J.I.); 2 Department Chemical Sciences and Materials Technology (DSCTM), National Research Council (CNR), Piazzale A. Moro 7, 00185 Roma, Italy; lidia.armelao@cnr.it; 3 Department Chemical Sciences, University of Padova, Via F. Marzolo 1, 35131 Padova, Italy

**Keywords:** ZrVTi, membrane, high-power impulse magnetron sputtering, multilayer, LCA, critical raw materials

## Abstract

Some metals belonging to groups IV and V show a high permeability to hydrogen and have been studied as possible alternatives to palladium in membranes for hydrogen purification/separation in order to increase their sustainability and decrease their costs. However, to date, very few alloys among those metals have been investigated, and no membrane studies based on 4–5 element alloys with low or zero Pd content and quasi-amorphous structure have been reported so far. In this work, new membranes based on ZrVTi- and ZrVTiPd alloys were tested for the first time for this application. The unprecedented deposition of micrometric-based multilayers was performed via high-power impulse magnetron sputtering onto porous alumina substrates. Dense Pd/Zr_x_V_y_Ti_z_Pd_w_/Pd multilayers were obtained. The composition of the alloys, morphology and structure, hydrogen permeance, selectivity, and resistance to embrittlement were tested and analyzed depending on the deposition conditions, and the membrane with the enhanced performance was tuned. The environmental impact of these membranes was also investigated to ascertain the sustainability of these alloys relative to more common Pd_77_Ag_23_ and V_93_Pd_7_ thin-film membranes using a life cycle assessment analysis. The results showed that the partial substitution of Pd can efficiently lead to a decrease in the environmental impacts of the membranes.

## 1. Introduction

Hydrogen is gaining renewed attention in Europe and around the world. As a raw material, fuel source, or energy carrier and storage medium, hydrogen has many possible applications in the sectors of industry, transport, and energy. By not emitting CO_2_ when used, it offers a solution to decarbonize industrial processes. However, today, hydrogen represents a modest fraction of the EU energy mix and is still largely produced from natural gas and fossil fuels [1].

Hydrogen production from fossil fuels is commonly performed via steam reforming, where an H_2_-rich gas mixture containing CO, CO_2_, and other by-products is obtained. Therefore, hydrogen must be purified for use in chemical production or fuel cells. In case of its use in blending with methane, an in-line separation process is also necessary. Membrane technology is currently increasingly considered a candidate for substituting conventional purification systems, owing to several advantages, including low energy consumption, ability to carry out separation continuously, simple scale-up, and on-site purification systems [2]. However, this technology will become increasingly attractive, particularly if membranes achieve low cost, and if critical raw materials are reduced in their production [3,4,5].

Most commercial membranes are based on palladium and its alloys. A reduction in costs and an increase in the economic and environmental sustainability of the membranes can be fostered by the identification of alternative metals to Pd, or by limiting the use of Pd to a few%. Some metals belonging to groups IV and V, and not included among the critical elements, show high permeability to hydrogen and have been studied as possible alternatives to Pd [4,5,6,7,8,9,10]. The rate of hydrogen transport through the lattice of group V metals is usually an order of magnitude higher than any other metallic lattice, including palladium [11].

Among group V metals, the high hydrogen solubility of vanadium at operational pressures may affect its mechanical stability due to embrittlement. To limit hydrogen solubility, various vanadium-based binary and ternary alloys have been tested, and Pd was identified as one of the most efficient alloying elements in substitutional alloys [4,5,6,7,8,9,10,11,12,13,14]. In particular, V_90_Pd_10_ and V_92.7_Pd_7.3_ led to promising results due to their optimal permeability to hydrogen and relativity good ductility [13,14,15]. Other vanadium alloys have only been investigated as hydrogen storage materials, as a range of AB2-type alloys (typically A = Ti, Zr and B = Cr, Mn, Ni, Fe, V) [16,17]. In 2013, Kunce et al. [18] reported a ZrTiVCrFeNi high-entropy alloy having the C14 structure, endowed with the ability to store hydrogen reversibly. In addition to AB2-type Laves phase structures such as those mentioned, BCC-type structures are also reported as in the work of Sahlberg et al. [19], in which a TiVZrNbHf alloy capable of dissolving hydrogen up to the concentration of H/M = 2.5 at 300 °C and 60 bar was studied. More recently, the properties of interactions with the hydrogen of TiVZr_z_NbTa_1−z_ and TiVZr_1+z_Nb high-entropy alloys have been reported by the Hauback group [20]. However, no membrane studies based on 4–5 element alloys with low or zero Pd content and quasi-amorphous structure (typical of high-entropy alloys) have been reported so far.

Naturally, this structural information can only give general guidance for identifying possible alloys for membranes. Another useful parameter in the alloy selection is the tendency to form hydrides, which must be sufficiently low (<20 kJ/mol H_2_ could be a reasonable value) to avoid membrane embrittlement at operating partial pressures and temperatures. In addition, semi-empirical principles can be applied to act specifically on stoichiometries, to improve or adjust the desired properties. For example, the maximum quantity of dissoluble hydrogen in a metal alloy is given by the number of interstitial sites for which the following two criteria are applicable: The distance between two hydrogen atoms occupying interstitial sites is at least 2.1 Å [21], and the sphere internal to the interstitial that touches all the first neighbors has a radius of at least 0.37 Å [22]. By acting according to these principles, and by changing the stoichiometries of the elements in the alloy and knowing the atomic radii, it is possible to modify the size and distances of the interstitial sites in order to adequately modify the hydrogen solubility. Another recently introduced semi-empirical method is the count of the medium valence electrons of the alloy (valence electron concentration (VEC)), which is the average of the valence electrons of each atom, weighted on the atomic percentages. On the basis of this value, the AB2 alloys tend, for example, to crystallize in the hexagonal C14 structure when the value is lower than 7 and cubic C15 if higher. Similarly, the hydride formation enthalpy and the hydrogen diffusion coefficient seem to be related to VEC [23,24]. On the basis of these semi-empirical methods and the literature data on hydrogen storage, various compositions of ZrVTi and ZrVTiPd alloys, never prepared and tested so far for membranes for hydrogen separation, were investigated in this study.

To further reduce the noble metal content, an effective strategy is to reduce dense selective layers to few µm films deposited onto porous substrates. Various Pd- and Pd-alloy-based membranes have been produced and tested as few µm films deposited onto porous alumina, nickel, or stainless steel [25], while other alloys such as vanadium-based alloy membranes have been tested mainly as sheets or tubes with thicknesses ≥ 40 µm [13,26], with only a few works on thin films [15]. These membranes need a protective coating against oxidation, and palladium is typically used with the further function of a catalytic layer for hydrogen absorption/desorption [4,13].

For the deposition of thin selective layers on top of porous substrates, magnetron sputtering has been demonstrated to be effective [15,27,28]. It also allows the sequential deposition of protective Pd layers and non-noble alloys in one stage in vacuum, thus avoiding oxidation at the interface. In this work, we exploited a recent evolution of this technique, high-power impulse magnetron sputtering (HiPIMS), for the deposition of a hydrogen-selective membrane film. HiPIMS combines magnetron sputtering with pulsed power technology, with the aim of generating highly ionized plasma with large quantities of ionized sputtered material [29]. The high degree of ionization of the sputtered species, combined with a bias voltage applied to the substrate, has been shown to lead to the growth of dense films with good control on composition and microstructure also onto porous substrates [15,29,30].

In this work, ZrVTi and ZrVTiPd films were deposited onto porous alumina using HiPIMS with variable compositions and tested as membranes for hydrogen permeation. Alumina was chosen as the material for porous substrates, as it is cheap and mechanically and chemically stable in operating conditions. Alumina substrates have also the advantage of preventing interdiffusion phenomena at high temperatures typically occurring with metallic substrates, a critical issue that can reduce hydrogen permeation.

The morphology, composition, and structure of membranes were characterized, and hydrogen permeability and selectivity were measured in the 300–450 °C range. In addition to the performances and features of the proposed new materials, it is pivotal to also consider the environmental sustainability aspects of palladium substitution. Therefore, a comparative life cycle impact assessment (LCA) was carried out with a “cradle-to-gate” approach, comparing the environmental impacts of the prepared membranes relative to more common PdAg-based systems.

## 2. Materials and Methods

### 2.1. Membrane Preparation

Alumina porous supports were prepared by mixing 35 vol% of α-Al_2_O_3_ (Alfa Aesar, Haverell, MA, USA, 99.9%) with 65 vol% of poly-methyl methacrylate powders as pore formers (PMMA, Soken Chemical & Engineering, Tokyo, Japan, average size 1.5 μm). A wet-ball milling process (absolute ethanol, Sigma-Aldrich (St. Louis, MI, USA) ACS reagent ≥ 99.8%) in zirconia jars was carried out in a planetary mill (Fritsch Pulverisette 7, Pittsboro, NC, USA) at 350 rpm for 2 h. The mixtures were uniaxially pressed (Nannetti Mignon SS/EA, Faenza, Italy) in a 2.5 cm diameter mold by a 140 MPa load. The disks were then sintered at 1500 °C in a high-temperature furnace (Nabertherm HT 04/17, Lilienthal, Germany), with an isotherm step of 1 h at the burning temperature of PMMA (386 °C) and a slow heating rate above 1000 °C (30 °C/h), when shrinkage is maximum, to avoid pellet bending. The sintered disks were then polished and cleaned in an ultrasonic bath. The porosity was estimated by measuring the geometrical density and comparing it with the theoretical value of 3.98 g/cm^3^ for full dense alumina. Planar and porous substrates were obtained, with ~2 cm diameter, 0.6–1 mm thickness, ~40% porosity, and mean surface pore sizes of about 500–600 nm confirmed by gas permeances measures [15].

The membranes were deposited via a combined DC/HiPIMS magnetron sputtering technique. Before each deposition, the chamber was evacuated to a base pressure ≤1 × 10^−4^ Pa, while depositions were conducted in an argon atmosphere (Ar, 99.999%) at 0.6 Pa. During depositions, the substrates were rotated at 5 rpm to improve homogeneity. Prior to alloy sputtering, a thin film (~300 nm) of Pd was deposited onto alumina at room temperature using HIPIMS (200 W). A co-sputtering process was developed for the ZrVTiPd alloy. Three targets were used: Pd, Zr, and an equimolar VTi alloy. The power on targets was properly regulated to achieve different compositions. The VTi target was powered by HiPIMS (Trueplasma HighPulse 4002, TRUMPF Hüttinger, Ditchingen, Germany) at a power ranging from 100 to 400 W, depending on the desired composition (i.e., between 5 and 20.4 W cm^−2^, pulse length 50 µs, and frequency 1000 Hz). The Zr target was powered by HiPIMS (Trueplasma HighPulse 4002, TRUMPF Hüttinger, Ditchingen, Germany) at a power ranging from 35 to 300 W depending on the desired composition (i.e., between 1.8 and 15.3 W cm^−2^, pulse length 50 µs, and frequency 1000 Hz), while Pd target was powered by DC (Trueplasma DC 4001, TRUMPF Hüttinger, Ditchingen, Germany) at 15 W (0.8 W cm^−2^). The target substrate distance was set at 100 mm. During depositions, various substrate heating temperatures were tested between 150 and 350 °C using IR lamps, and two negative bias voltages were tested, i.e., 100 and 125 V (Trueplasma Bias 3018, TRUMPF Hüttinger, Ditchingen, Germany). After alloy deposition, only the Pd deposition was continued to produce a pure Pd thin film (~300 nm) on the top surface.

### 2.2. Characterization

Structural and microstructural information on the crystalline phases of the membranes were obtained via XRD and Rietveld refinement. The patterns were recorded at room temperature using a Bruker D8 ADVANCE Plus diffractometer with Bragg–Brentano geometry, employing a Cu anode X-ray tube operated at 40 kV and 30 mA (angular range from 20° to 90°; 0.02° step; 6 s per step). Rietveld refinements on X-ray powder diffraction patterns were performed using MAUD software [31]. Surface and fractured surfaces of samples were observed via field-emission scanning electron microscopy (FE-SEM) with a SIGMA Zeiss instrument (Zeiss Microscopy GmbH Germany), operating in high-vacuum conditions at an accelerating voltage of 20 kV, and the composition was determined via energy-dispersive spectroscopy (EDS, X-MAX, Oxford Instruments, UK).

Membrane permeability measurements were carried out by means of a custom-built stainless steel test station [15]. Membranes were clamped and sealed in a stainless-steel module using graphite gaskets. The module consists of two parts, the feed side and the permeate side, which were connected by a channel of 1 cm in diameter, closed by the membrane to be tested, and placed in a furnace (Nabertherm N11/HR). The membrane housing temperature was monitored using a K-type thermocouple inserted directly in the module test. The gas flows at the feed and permeate sides were set using independent mass flow controllers (1179A, 1179B, and 647C, MKS). The pressure at the feed side was controlled with a Baratron pressure transducer (722B, MKS). Nitrogen (99.999%) was used to test membrane selectivity, while high-purity hydrogen was produced using an electrolyzer (PerkinElmer PGX Plus H2 160, Singapore).

During permeability tests, the feed-side pressure varied from atmospheric pressure to 400 kPa, while on the permeate side, it was maintained at atmospheric pressure by a sweep gas flow. The gas flow on the feed side was measured after reaching stationary conditions (flow fluctuations < 1%). Pressure, flow rates, and temperatures were controlled and monitored using a LabVIEW interface. After reaching stationary conditions, hydrogen permeation was measured in the 300–450 °C range and from 1 to 300 kPa of the pressure difference between feed and permeate sides. The measures were repeated for membranes deposited in the same conditions to verify repeatability. The selectivity was evaluated by the ratio between hydrogen and nitrogen permeances.

### 2.3. Life Cycle Assessment

A life cycle assessment (LCA) model was built on the sole environmental impacts related to the metal elements of the membranes showing sufficient stability to hydrogen permeation. Considering a comparison among membranes prepared with the same processes, the substrates, production processes, infrastructures, labor, instrumentations, and end-of-life stages between the different kinds of membranes were deemed similar and, thus, neglected when performing a comparative LCA. Gas selectivity and the different mechanical and performance stabilities of the considered membranes were considered outside the system boundaries. Membranes with Pd_77_Ag_23_ and V_93_Pd_7_ compositions presented in previous works and analogously prepared onto the same substrates [15,28] were included in our analysis to evaluate just the impact contribution of alloy composition.

The activity data associated with the life cycle inventory of membrane elements were mainly retrieved from Ecoinvent 3.7.1 database. Metallic vanadium and zirconium were supposed to be produced using the Kroll method, a pyrometallurgical industrial process mainly used to produce metallic Ti from TiCl4 [32,33] and, thus, adapted from the process”titanium primary, triple-melt {GLO}|titanium production, primary, triple melt|cut-off, U” (Tables S6 and S8).

The VCl_3_ activity data were adapted from part of the inventory process for the battery vanadium electrolyte reported in the literature [34] (a complete inventory of the processes is listed in Tables S1–S6 of the Supplementary Materials).

Two assessments were performed: in the first case, the functional unit of the analyzed system was set as the same mass quantities (e.g., 1 kg) of the metal component of each membrane for a preliminary analysis.

In the second case, the functional unit was identified as hydrogen permeance of the considered membranes. Thus, with the aim of comparing the environmental impacts of membranes with different permeance rates, virtual coating thicknesses were set to match the permeability of 1 kg of Pd_77_Ag_23_ taken as a reference. Exploiting the theoretical densities of the film crystalline phases, the respective mass ratio of the membranes was calculated, thus obtaining the reference flow used for the life cycle impact assessment (LCIA).

LCIA was carried out by employing SimaPro 9.2 software, using the environmental footprint method, as recommended by the European Commission [35]. The complete Life cycle assessment inventory is reported in the Supplementary Materials (Tables S1–S14).

## 3. Results and Discussion

With the intention of completely or partially replacing palladium from metal membranes, to date, some metals and alloys of the group V elements have been investigated as bulk, but much remains to be studied regarding alloys with more components or high-entropy alloys. Reducing the metal content by depositing these alloys as thin films is still a matter of research. From this perspective, many alloys were studied in this work. For all of the studied alloys, the deposition process was optimized to obtain dense films onto porous alumina by means of HIPIMS, and their performances were tested. In particular, VNi and VNiPd (deposited by two or three targets of the individual elements), FeTi and FeTiPd (from one target of FeTi and one of Pd), CrFeNiTi (from one target of CrFeNi and one of Ti), and NbZrVTi (from one target of Nb and one of ZrVTi) were tested. However, all membranes prepared using these alloys showed hydrogen embrittlement at exposure to a hydrogen atmosphere. The only alloys that were found to be interesting are ZrVTi and ZrVTiPd in various compositions.

### 3.1. Membrane Development

The production of membranes was based on a selective few µm thick-layer film made of ZrVTi alloys with low palladium content, which were protected from oxidation by two palladium thin films (300 nm) and deposited onto porous alumina, and the process was performed in a few steps. A first Pd layer was preliminarily deposited onto a porous alumina substrate (at room temperature, via unbalanced DC, a deposition rate of 35–60 nm/min, and a Pd thickness of about 0.3–4 µm) in order to create a conductive layer to improve bias application during the alloy deposition and to protect the selective metallic layer from oxidation.

HiPIMS depositions of ZrVTi and ZrVTiPd alloys onto Pd/alumina substrates were then carried out for up to 5 h. Mean alloy deposition rates between 0.9 and 1.3 µm/h, depending on alloy composition, were calculated, and alloy thickness ranging from about 4 to 7 µm were measured. Various compositions were prepared. The multilayer was then completed with a top layer of Pd (thickness of about 0.3–4 µm). The two Pd thin films in the multilayer membranes have multiple roles and purposes: to protect alloy from oxidation, to prevent interdiffusion phenomena, and to act as catalytic layers for hydrogen splitting/recombination. The total thickness of the multilayer films was between about 5 and 8 µm. Table 1 reports an overview of some representative deposition conditions tested and the corresponding thickness and alloy composition measured via EDS.

**Figure 1:**
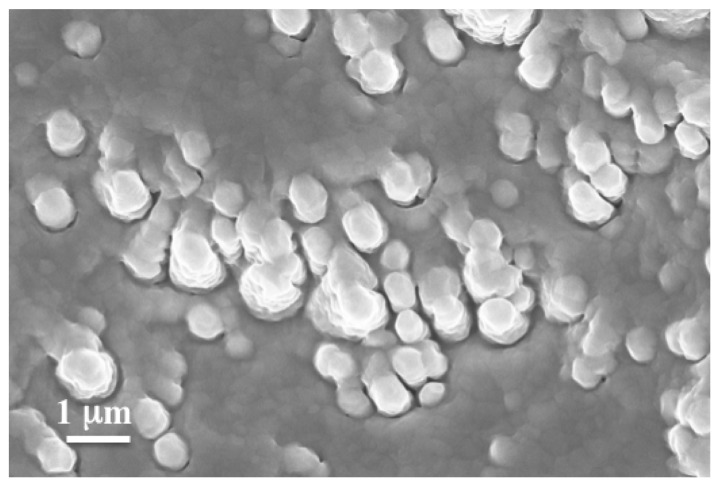
Surface SEM micrograph of ZrVTi1 sample deposited at 350 °C.

According to the Thornton model [36], the microstructure and crystallinity of PVD thin films should improve when the deposition temperature approaches the melting temperature of the deposited material. However, in the case of this alloy, if the temperature was maintained at 350 °C during deposition, large crystal formation with a dendritic-like morphology resulted in films with high roughness and microporosity (see Figure 1).

**Figure 2:**
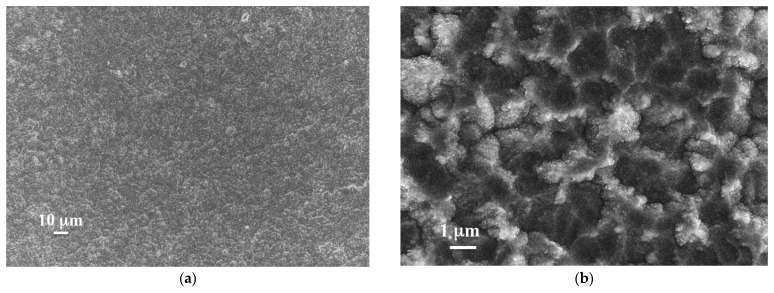
Surface SEM micrographs at different magnifications of the Pd top surface for ZrVTi2 (**a**) and ZrVTiPd3 (**b**) samples.

Conversely, with the temperature set at 150 °C during deposition, membranes showed homogeneous, compact, and dense films, as revealed by SEM micrographs of the top surface. No pores or defects were detected. Figure 2 reports two representative surface SEM micrographs of ZrVTi2 and ZrVTiPd3 samples.

**Figure 3:**
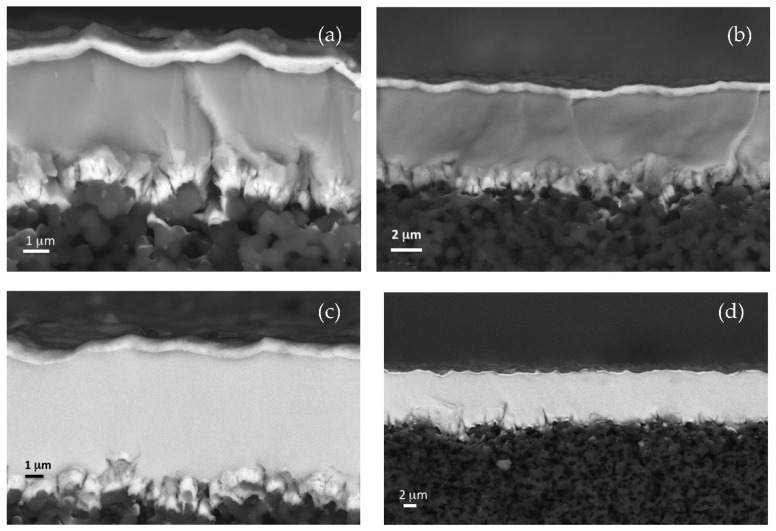
Some representative cross-sectional view of ZrVTi1 (**a**), ZrVTiPd1 (**b**), ZrVTiPd3 (**c**), and ZrVTiPd5 (**d**) membranes, taken under backscattered electron mode.

Observing the cross-section of samples, a very compact, dense, and smooth metallic alloy film was detected between the two thin palladium films (see Figure 3, in which some representative cross-sectional views of membranes are reported). Figure 3 shows how the HIPIMS technique was effective in achieving a compact film even on top of the porous and insulating alumina substrate. The backscattered electron images also highlight the two thin palladium films on top and at the metal/alumina interface.

**Figure 4:**
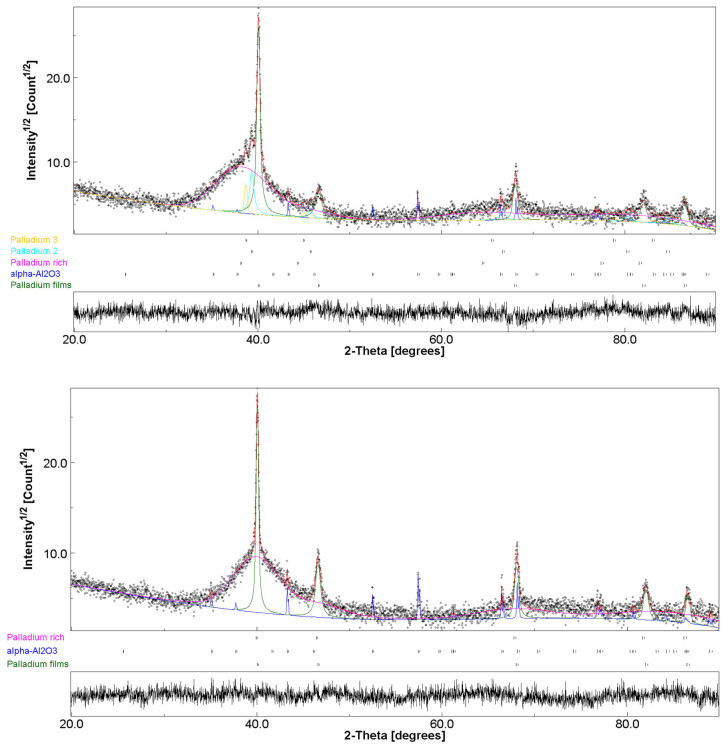
XRD patterns and Rietveld refinements of ZrVTiPd4 (**up**) and ZrVTiPd6 (**down**) samples.

XRD analyses of only the alloy without the thin Pd film on top were not performed due to oxide presence. Figure 4 shows two typical XRD profiles collected for the ZrVTiPd4 sample prior to hydrogen permeation tests and for ZrVTiPd6 after the tests. Rietveld refinements were performed by taking into account and modeling the systems as multilayers composed of porous α-Al_2_O_3_ and supporting the three thin layers made as Pd/Zr_x_V_y_Ti_z_Pd_w_/Pd. The fit of the ZrVTiPd4 sample had a weighted profile R-factor (Rwp) of 21.0% and a goodness-of-fit (χ^2^) value of 1.18, while the ZrVTiPd6 sample had Rwp = 21.8% and χ^2^ = 1.17. Both profiles showed the small sharp peaks of the highly crystalline, porous α-Al_2_O_3_ (trigonal, space group R-3c), showing exactly the same cell parameters (a = 4.763 ± 0.001 Å, c = 13.007 ± 0.001 Å) in all samples. The protective films contributed with the most intense (111) peak at about 40.05° for all samples, corresponding to pure Pd (cubic, space group Fm-3m) with the same cell parameters of 3.895 ± 0.001 Å. The ZrVTiPd4 sample showed small contributions of two Pd-like phases with (111) peaks at 38.7° and 39.3°, probably arising from the solid solution of atoms at the interface, especially due to Ti and Zr that have larger atomic radii than Pd. The intermediate alloy film exhibited as a quasi-amorphous phase in both cases and generally in all considered membranes, with a broad band between 35° and 45°. The contribution was modeled with a Pd-like phase in which cell parameter was influenced by the presence of Zr, Ti, and V in a solid solution. The cell parameter was a = 4.08 Å for the ZrVTiPd4 sample and a = 3.89 Å for ZrVTiPd6. The values were consistent with the considerably higher concentration of Zr in the ZrVTiPd4 sample, which had a larger atomic radius than Ti, V, and Pd. The presence of the quasi-amorphous alloy in the as-deposited films, which was also confirmed in the spectra of films treated at 350 °C and in membranes tested in hydrogen up to 450 °C, demonstrated the microstructural stability of the membranes. The amorphous nature of multicomponent alloys has often been observed in high-entropy alloys [37].

### 3.2. Functional Characterization

The functional characterization analysis started with the nitrogen flux measurements at temperatures ranging from 300 to 450 °C and with a pressure difference from the feed to the permeate side that ranged between 10 and 300 kPa. Then, the hydrogen fluxes were measured in the 300–450 °C range as a function of the pressure difference between the feed and the permeate side starting from very low ∆P (1–2 kPa).

Prior to membrane characterization, the alumina porous substrates were tested by measuring the nitrogen and hydrogen fluxes in order to verify the mass transfer resistance in the substrate. The ratio between the resistance to flux in the membrane (*R_mem_*) and that to the sole substrate (*R_sub_*) is:

(1) $$\frac{R_{mem}}{R_{sub}}=\frac{\frac{1}{J_{mem}}}{\frac{1}{J_{sub}}}$$

where *J* is the measured flux, which may give a qualitative indication of the mass transfer resistance in the substrate. This value is close to 80 and typically indicates the presence of a low mass transfer resistance contribution due to the porous substrate [38], and it was considered in the evaluation of the membrane flux. Permeance measurements in the substrate also allowed an evaluation of the average porosity, as already reported by Fasolin et al. [15].

The performance of membranes strongly depended on the alloy composition. When the zirconium content was >60 at%, films with apparent high stresses were produced, and the membranes broke in a nitrogen atmosphere, when a ∆P of 300 kPa at 450 °C was applied, as in the case of the ZrVTiPd5 sample, while other membranes were stable in a nitrogen atmosphere.

In a hydrogen atmosphere, all membranes with Pd content ≤ 21 at% experienced hydrogen embrittlement even with a small pressure difference between the two sides (see Table 2). At higher Pd content, the resistance to embrittlement increased until resistance to 300 kPa of pressure difference in the case of a 39 at%Pd content (see Table 2). As expected, the permeances decreased with the increase in embrittlement resistance, reducing from a maximum of 8.1 × 10^−6^ mol m^−2^ s^−1^ Pa^−1^ to a minimum of 2.0 × 10^−7^ mol m^−2^ s^−1^ Pa^−1^.

**Figure 5:**
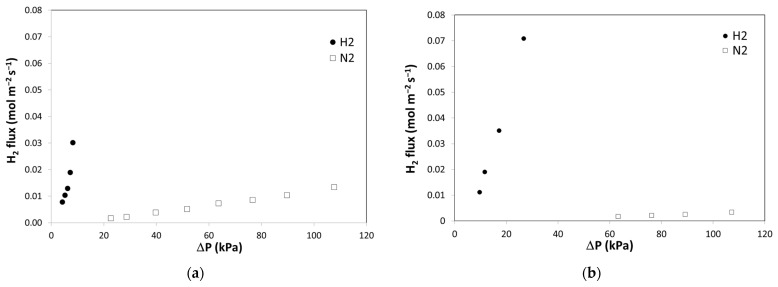
Hydrogen and nitrogen fluxes measured in ZrVTiPd2 (**a**) and ZrVTiPd3 (**b**) as a function of the pressure difference between feed and permeate sides.

The flux values for some of the membranes resulted in extremely high values, considering the low ∆P and the high selectivity that reached the maximum level allowed by the testing apparatus. As shown in Figure 5, hydrogen and nitrogen fluxes measured in ZrVTiPd2 (a) and ZrVTiPd3 (b) membranes are reported as a function of the pressure difference between the feed and the permeate side. Hydrogen flux values reached values around 0.07 mol_H2_ m^−2^ s^−1^ at ∆P = 25 kPa. These values are 3 times higher than those reported for the Pd/V_93_Pd_7_/Pd multilayer at the same ∆P [15]. However, these values are due to high hydrogen solubility in the alloy, which, however, also reduced the operating conditions to low ∆P due to the tendency to embrittlement at higher ∆P. Various experiments were conducted to vary the composition of the alloy and optimize this resistance to embrittlement. High resistance to hydrogen embrittlement was reached, for example, for the ZrVTiPd6 membrane but at the expense of hydrogen solubility. In fact, the ZrVTiPd6 membrane showed lower hydrogen permeance and selectivity, indicating a lower diffusion of hydrogen in the alloy.

By investigating the trend of flux with pressure, the trend with ($\sqrt{P_{feed}}-\sqrt{P_{perm}})$, according to Sievert’s law, was not detected, as already observed for thin palladium-based membranes [39]. A general formula is typically used to estimate permeability (Φ) or permeance (Φ/*L*) of thin film-based membranes, defined in Equation (2) [4]:

(2) $$J=\frac{\Phi }{L}\left(P_{feed}^{n}-P_{perm}^{n}\right)$$

where *L* is film thickness. Although limited, this formula represents a general approach to consider some phenomena that can lead to the best fit of flux data vs. (*P^n^_feed_* − *P^n^_perm_*) with an exponent *n* higher than 0.5. Among them, there are surface resistance to H_2_ absorption and desorption, mass transfer resistance in porous substrates, hydrogen diffusion as molecular H_2_ along grain boundaries, and/or Knudsen or viscous flow through pores and film defects and leaks [4,40,41]. In thin Pd membranes, *n* was shown to approach 1, and this is typically attributed to surface phenomena and/or to a contribution of Knudsen flow [39].

For all membranes investigated in this work, an almost linear trend with ∆*P* (*n* = 1) was observed. This behavior is due to a combination of surface- and interface-controlled phenomena, hydrogen solubility in the alloy, and mass transfer resistance in the porous supports on the low-pressure side. However, considering that the apparent activation energies were found to be not significantly influenced by surface phenomena, the best fit with *n* = 1 seemed to confirm the hypothesis that the flux in these membranes is predominantly controlled by a combination of a diffusion-limited process over the alloy and a mass transfer resistance in the porous support. The permeance values reported in Table 2 were calculated with *n* = 1. If comparing the permeance values with thin palladium-based membranes [39], the permeance values were similar to palladium-based membranes and with a similar trend (*n* = 1), even though the stability was much lower.

By analyzing the trend of the flux as a function of temperature, an increase in flux values was observed with the increase in temperature for samples with high permeance values. For example, in the ZrVTiPd2 sample, the permeance values changed from 2.55 × 10^−6^ at 300 °C to 3.78 × 10^−6^ mol m^−2^ s^−1^ Pa^−1^ at 400 °C. By extrapolating the data at various temperatures, the apparent activation energy calculated, e.g., for the ZrVTiPd2 membrane was 12.8 kJ mol_H_^−1^. This value is consistent with the typical values reported for the activation energy of hydrogen diffusion in some metals such as vanadium [4]. In fact, the typical activation energy for diffusion-limited flow in Pd is estimated to be much higher (around 22–24 kJ mol_H_^−1^) [4,42]. Therefore, permeation seems kinetically controlled by the ZrVTiPd layer instead of Pd thin films, surface phenomena, or desorption-limited processes, which have significantly higher activation energies (>42 kJ mol_H_^−1^).

Conversely, when the palladium content increased in these alloys, the hydrogen diffusion strongly reduced, and the trend with temperature changed. In fact, for the ZrVTiPd6 sample, there was a decrease in flux with temperature (e.g., decreased from 2.67 × 10^−7^ at 350 °C to 2.00 × 10^−7^ mol m^−2^ s^−1^ Pa^−1^ at 400 °C). This trend and the low selectivity for this membrane are in line with a Knudsen-type flux.

**Figure 6:**
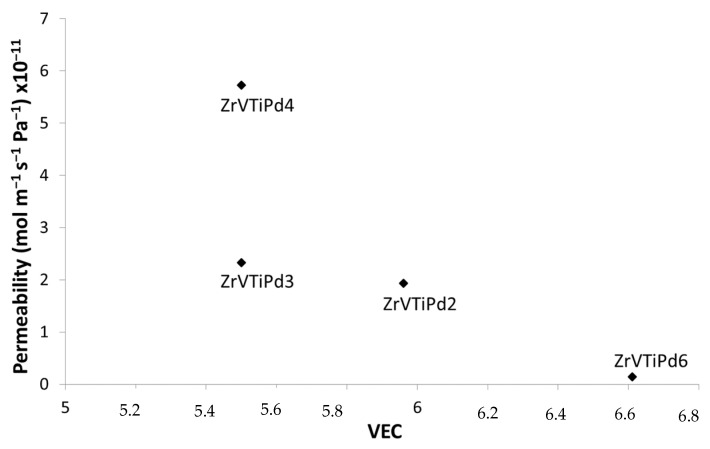
Hydrogen permeability measured at 350 °C for some membranes as a function of VEC.

If multielement alloys are considered, as in this case, the hydrogen absorption and diffusivity are known to be dependent on valence electron concentration (VEC, Equation (3)):

(3) $$\mathrm{VEC}=\sum_{i=1}^{N}\left\{c_{i}\right.\left.{\left(\mathrm{VEC}\right)}_{i}\right\}$$

where *c_i_* is the atomic fraction of element *i* with valence electron concentration (VEC)*_i_*. Figure 6 reports the hydrogen permeability at 350 °C for various membranes as a function of VEC [43].

Figure 6 shows a trend of permeability that decreased with VEC. The reduction trend is in line with a reduction in hydrogen solubility with VEC already reported in other works on high-entropy alloys [43]. Therefore, this parameter could be used to tune other alloy compositions.

**Figure 7:**
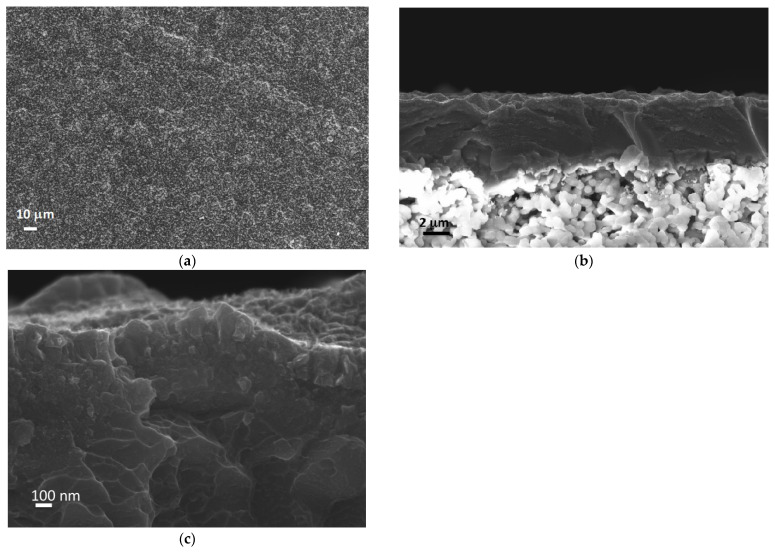
Surface (**a**) and cross-sectional (**b**) SEM micrographs of the ZrVTiPd6 membrane after permeability tests and a particular (**c**) of the surface Pd thin film.

The morphological characterization of membranes after permeability tests in hydrogen (Figure 7) indicated that they retained a homogeneous surface. The cross-section showed homogeneous films without cracks or defects and crystal growth in the thin palladium surface film. XRD analyses after tests (Figure 4) showed spectra analogous to spectra of as-deposited films, indicating the stability of film structure during tests.

### 3.3. Comparative Life Cycle Assessment Analysis

To develop membranes with reduced environmental impact compared with those based on Pd, a comparison of the respective load of metal membranes was made between those showing promising permeance and selectivity values, observed during functional characterization (Table 3).

**Figure 8:**
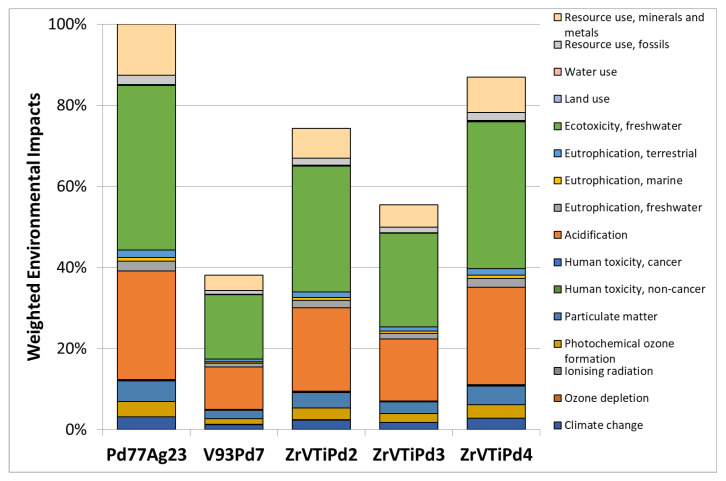
Comparison of weighted environmental impacts of the same mass quantity of different membrane compositions and Pd contents relative to Pd_77_Ag_23_.

A preliminary comparative LCIA analysis was performed considering the same mass quantity of the membranes with different compositions, taking the Pd_77_Ag_23_ weighted environmental impact as a reference (Figure 8).

The main contributions to the total environmental impacts were related to impact categories of “resource use, minerals, and metals” (10–13% of impact weight), “ecotoxicity, freshwater” (41–42%), and “acidification” (27–28%).

The Pd content clearly affected the total environmental impacts. Indeed, the less impacting membrane for hydrogen separation was found to be V_93_Pd_7_, possessing a lower Pd content (weighted environmental impact decrease of 62%), followed by ZrVTiPd3 (45% reduction).

If the performance was compared, the weighted environmental impacts were rescaled as a function of functional characterization. Since the permeability can be calculated by multiplying permeance per membrane thickness, virtual membrane thicknesses were set to obtain the same Pd_77_Ag_23_ permeability, measured at 350 °C. This estimation can be considered allowed only in the case in which the thickness variation is small enough not to invalidate the permeability model used (see Section 3.2. Functional Characterization). Therefore, the respective mass ratio of the membranes was calculated using the theoretical densities of the membrane crystalline phases, obtaining the reference flow used for the life cycle impact assessment (see the values listed in Table 3).

**Figure 9:**
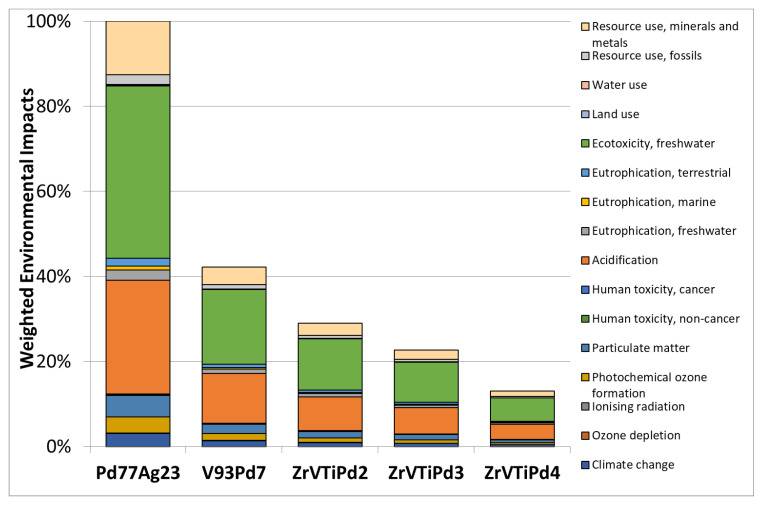
Comparison of weighted environmental impacts of different membrane compositions and Pd contents relative to Pd_77_Ag_23_, rescaled by the material permeance performance.

Figure 9 illustrates the comparison of the weighted environmental impacts of the considered membranes, with the same calculated hydrogen permeability.

The main contributions to the total environmental impacts of the membranes remained the same as those in the previous LCA analysis (i.e., “resource use, minerals, and metals”; “ecotoxicity, freshwater”; and “acidification”), maintaining the same impact contributions. Nevertheless, rescaling membrane mass quantities as a function of their permeance performances affected the values of the total environmental impacts, changing the landscape of the results. In this configuration, ZrVTiPd4 showed an impact decrease of 87%, compared with PdAg, hence the most environmentally sustainable membrane for hydrogen separation within the system boundary conditions considered. In fact, it is worth underlining that although ZrVTiPd4 was the membrane with the lowest environmental impact, its mechanical stability under operating conditions must be improved for it to be used in practical applications.

## 4. Conclusions

The replacement of or the reduction in palladium in metal membranes for hydrogen separation is an inevitable and crucial step in the development of future membranes, to increase their environmental sustainability and reduce costs, also given the considerable recent increases in palladium cost. With this view in mind, in this study, a possible alternative was investigated by preparing more sustainable membranes based on ZrVTi and ZrVTiPd alloys. In particular, various alloy compositions were investigated and deposited via HiPIMS onto porous alumina substrates. Dense Pd/Zr_x_V_y_Ti_z_Pd_w_/Pd multilayers were obtained. The identification of the optimal composition was a challenging process. Compositions with high zirconium content proved unsuitable, as they were not stable under operating conditions. Conversely, compositions with good hydrogen permeance values (up to 8.07 × 10^−6^ mol m^−2^ s^−1^ Pa^−1^) were identified but with resistance to embrittlement limited to low drops in pressure. A composition was also identified with high resistance to hydrogen embrittlement (up to 300 kPa of pressure difference) but with limited hydrogen solubility. The life cycle assessment analysis verified the environmental sustainability of the employed materials, demonstrating that the partial substitution of Pd can substantially decrease the environmental impacts. Two configurations were analyzed in comparative LCA, and in both cases, the membranes based on ZrVTiPd alloys resulted in having a lower impact than that of a reference PdAg membrane analogously prepared. This set of results confirmed the potential of these membranes as valid alternatives to more common Pd or PdAg alloys. The present study provides some basic reference data for the further development of hydrogen-permeable ZrVTi membranes.

## Figures and Tables

**Table 1:** Deposition conditions and the corresponding thickness and alloy composition measured via EDS on some representative membranes.

Sample Name	VTi Target Power (W)	Zr Target Power (W)	Pd Target Power (W)	Multilayer Thickness (µm)	Composition (at%)
ZrVTi1	400	100	--	5.4	Zr 35
					V 34
					Ti 31
ZrVTi2	400	35	--	5.3	Zr 21
					V 42
					Ti 37
ZrVTiPd1	400	100	15	7.4	Zr 19
					V 36
					Ti 26
					Pd 19
ZrVTiPd2	400	35	15	5.3	Zr 9
					V 34
					Ti 30
					Pd 27
ZrVTiPd3	130	260	15	6.7	Zr 52
					V 12
					Ti 13
					Pd 23
ZrVTiPd4	250	130	15	7.1	Zr 39
					V 20
					Ti 19
					Pd 22
ZrVTiPd5	100	300	15	7.8	Zr 61
					V 9
					Ti 9
					Pd 21
ZrVTiPd6	400	35	20	5.2	Zr 11
					V 27
					Ti 23
					Pd 39

**Table 2:** Hydrogen permeance, selectivity, and embrittlement resistance values for some membranes (the composition is also reported to ease the comparison).

Sample	Composition (at%)	Permeance (mol m^−2^ s^−1^ Pa^−1^)	Hydrogen Embrittlement	Selectivity (Permeance H_2_/Permeance N_2_)
ZrVTi1	Zr 35 V 34 Ti 31		∆P~0 kPa	
ZrVTi2	Zr 21 V 42 Ti 37		∆P~0 kPa	
ZrVTiPd1	Zr 19 V 36 Ti 26 Pd 19		∆P~0 kPa	
ZrVTiPd2	Zr 9 V 34 Ti 30 Pd 27	3.49 × 10^−6^ at 350 °C	∆P > 10 kPa	50
ZrVTiPd3	Zr 52 V 12 Ti 13 Pd 23	3.48 × 10^−6^ at 350 °C	∆P > 30 kPa	100
ZrVTiPd4	Zr 39 V 20 Ti 19 Pd 22	8.07 × 10^−6^ at 300 °C	∆P > 5 kPa ∆P~0 kPa for T > 300 °C	75
ZrVTiPd5	Zr 61 V 9 Ti 9 Pd 21		∆P~0 kPa	
ZrVTiPd6	Zr 11	2.67 × 10^−7^ at 350 °C	∆P > 300 kPa	5
	V 27			
	Ti 23			
	Pd 39			

**Table 3:** Reference flow used for the life cycle impact assessment (membrane mass ratios calculated relative to a Pd_77_Ag_23_ membrane prepared by the same method; permeances measure at 350 °C).

Sample	Permeance (mol m^−2^ s^−1^ Pa^−1^)	Quantity (kg)
Pd_77_Ag_23_	1.8 × 10^−6^	1
V_93_Pd_7_	9.0 × 10^−7^	1.11 ^a^
ZrVTiPd2 (Zr_9_V_34_Ti_30_Pd_27_)	3.5 × 10^−6^	0.39 ^a^
ZrVTiPd3 (Zr_52_V_12_Ti_13_Pd_23_)	3.5 × 10^−6^	0.41 ^a^
ZrVTiPd4 (Zr_39_V_20_Ti_19_Pd_22_)	8.1 × 10^−6 b^	0.15 ^a^

^a^ For these membranes, where two thin films of palladium were used to protect the alloy, a total Pd film thickness of 600 nm was considered. ^b^ For this membrane, the permeance at 300 °C was considered.

## Data Availability

Not applicable.

## Supplementary Materials

The following supporting information can be downloaded at: https://www.mdpi.com/article/10.3390/membranes12070722/s1, Table S1: Life cycle inventory for vanadium bearing magnetite. Table S2: Life cycle inventory for Vanadium pentaoxide V_2_O_5_ bearing cast iron. Table S3: Life cycle inventory for Vanadium Slag (25% V_2_O_5_): Table S4: Life cycle inventory for Vanadium Pentaoxide V_2_O_5_. Table S5: Life cycle inventory for Vanadium Cloride VCl_3_. Table S6: Life cycle inventory for Vanadium. Table S7: Life cycle inventory for Zirconium tetrachloride. Table S8: Life cycle inventory for Zirconium. Table S9: Life cycle inventory for Pd_77_Ag_23_ membrane. Table S10: Life cycle inventory for V_93_Pd_7_ membrane with Pd coatings. Table S11: Life cycle inventory for Ti_30_Zr_9_V_34_Pd_27_ membrane (ZrVTiPd2) with Pd coatings. Table S12: Life cycle inventory for Ti_13_Zr_52_V_12_Pd_23_ membrane (ZrVTiPd3) with Pd coatings. Table S13: Life cycle inventory for Ti_19_Zr_39_V_20_Pd_22_ membrane (ZrVTiPd4) with Pd coatings. Table S14: Life cycle inventory for Ti_23_Zr_11_V_27_Pd_39_ membrane (ZrVTiPd6) with Pd coatings.
