# Supplementary material for: New Sustainable Multilayered Membranes Based on ZrVTi for Hydrogen Purification
Source: Membranes (Basel). 2022 Jul 21;12(7):722. doi: 10.3390/membranes12070722 (PMC9317777; doi:10.3390/membranes12070722)
Supplement: Supplementary file 1 [file membranes-12-00722-s001.zip › membranes-1814822-supplementary.pdf]

New Sustainable Multilayered Membranes Based on ZrVTi for Hydrogen Purification

Stefano Fasolin [1], Simona Barison [1,*], Filippo Agresti [1], Simone Battiston [1], Stefania Fiameni [1], Jacopo Isopi [1], and Lidia Armelao [2,3]

[1] Institute of Condensed Matter Chemistry and Technologies for Energy (ICMATE), National Research Council (CNR), Corso Stati Uniti 4, 35127 Padova, Italy; stefano.fasolin@cnr.it (S.F.); filippo.agresti@cnr.it (F.A.); simone.battiston@cnr.it (S.B.); stefania.fiameni@cnr.it (S.F.); jacopo.isopi@icmate.cnr.it (J.I.)

[2] Department Chemical Sciences and Materials Technology (DSCTM), National Research Council (CNR), Piazzale A. Moro 7, 00185 Roma, Italy; lidia.armelao@cnr.it

[3] Department Chemical Sciences, University of Padova, Via F. Marzolo 1, 35131 Padova, Italy

* Correspondence: simona.barison@cnr.it; Tel.: +39-0498295855

Supplementary Materials

The complete Life cycle assessment inventory is listed in the following tables (tables S1-S14).

Table S1: Life cycle inventory for vanadium bearing magnetite (adapted from Da Silva et al 2021 [35]).

Dataset	Amount	Unit
**Inputs**		
Occupation, mineral extraction site	0.00249	m^2a
TiO_2, 54% in ilmenite, 18% in crude ore	0.25	kg
Iron ore	1.08	kg
Transformation, from forest, unspecified	0.000083	m^2
Transformation, to mineral extraction site	0.000083	m^2
Vanadium ore	0.034	kg
Water, well, GLO	0.000059	m^3
Blasting {GLO}| market for | Cut-off, U	0.00015	kg
Conveyor belt {GLO}| market for | Cut-off, U	5.6E-08	m
Diesel, burned in diesel-electric generating set, 10MW {GLO}| market for | Cut-off, U	0.0238	MJ
Electricity, medium voltage {CN}| market group for | Cut-off, U	0.0144	kWh
Heat, central or small-scale, natural gas {GLO}| market group for | Cut-off, U	0.0154	MJ
industrial machine, heavy, unspecified {RoW}| market for industrial machine, heavy, unspecified | Cut-off, U	0.000012	kg
Mine infrastructure, open cast, ilmenite from hard-rock ore {GLO}| market for | Cut-off, U	3.1E-11	p
Petrol, unleaded {RoW}| market for | Cut-off, U	0.0627	kg
Recultivation, ilmenite mine {GLO}| market for | Cut-off, U	0.000083	m^2
**Outputs**		
Vanadium bearing magnetite (Magnetite 72% Fe, 2.2% V_2O_5)	1.53	kg
Ilmenite, 54% titanium dioxide	0.46	kg

Particulates, < 2.5 um to air	0.000018	kg
Particulates, < 10 um to air	0.00024	kg
Particulates, > 2.5 um, and < 10um to air	0.000096	kg
Water to air	0.00879	kg
Water, GLO to water	0.00005	m^3

Table S2: Life cycle inventory for Vanadium pentaoxide V_2O_5 bearing cast iron (adapted from Da Silva et al 2021 [35]).

Dataset	Amount	Unit
**Inputs**		
Water, cooling, unspecified natural origin, RoW	0.005216	m^3
Anode, for metal electrolysis {GLO}| market for | Cut-off, U	0.003	kg
Electric arc furnace converter {GLO}| market for | Cut-off, U	4E-11	p
Hard coal {CN}| market for | Cut-off, U	0.014	kg
Natural gas, high pressure {RoW}| market for | Cut-off, U	0.025	m^3
Oxygen, liquid {RoW}| market for | Cut-off, U	0.05073	kg
Quicklime, in pieces, loose {RoW}| market for quicklime, in pieces, loose | Cut-off, U	0.055	kg
Refractory, basic, packed {GLO}| market for | Cut-off, U	0.0135	kg
Pre-reduced V_2O_5 magnetite adapted from Iron pellet {RoW}| production | Cut-off, U	1.46	kg
Electricity, medium voltage {GLO}| market group for | Cut-off, U	0.42	kWh
**Outputs**		
Vanadium pentaoxide V_2O_5 bearing cast iron	1.320	kg
Benzene to air	2.31E-06	kg
Cadmium to air	3.65E-08	kg
Carbon monoxide, fossil to air	0.00232	kg
Chromium to air	1.25E-06	kg
Copper to air	2.31E-07	kg
Dioxins (TEQ) to air	4.54E-12	kg
Hydrocarbons, aromatic to air	7.7E-05	kg
Hydrogen chloride to air	5.2E-06	kg
Hydrogen fluoride to air	2.35E-06	kg
Lead to air	1.81E-06	kg
Mercury to air	2.22E-06	kg
Nickel to air	7.01E-07	kg
Nitrogen oxides to air	0.00018	kg
PAH, polycyclic aromatic hydrocarbons to air	3.73E-08	kg
Particulates, < 2.5 um to air	0.000166	kg
Particulates, > 10 um to air	5.86E-05	kg
Particulates, > 2.5 um, and < 10um to air	1.6596	kg
Polybrominated biphenyls to air	2.33E-08	kg
Sulfur dioxide to air	0.000077	kg

Water to air	0.002021	kg
Zinc to air	2.29E-05	kg
Inert waste, for final disposal {RoW}| market for inert waste, for final disposal | Cut-off, U	0.005	kg
electric arc furnace dust {RoW}| market for electric arc furnace dust | Cut-off, U	0.0096	kg
electric arc furnace slag {RoW}| market for electric arc furnace slag | Cut-off, U	0.928	kg

Table S3: Life cycle inventory for Vanadium Slag (25% V_2O_5) (adapted from Da Silva et al 2021 [35]).

Dataset	Amount	Unit
**Inputs**		
Water, cooling, unspecified natural origin, RoW	0.005216	m^3
Anode, for metal electrolysis {GLO}| market for | Cut-off, U	0.003	kg
Electric arc furnace converter {GLO}| market for | Cut-off, U	4E-11	p
Hard coal {CN}| market for | Cut-off, U	0.014	kg
Natural gas, high pressure {RoW}| market for | Cut-off, U	0.025	m^3
Oxygen, liquid {RoW}| market for | Cut-off, U	0.05073	kg
Quicklime, in pieces, loose {RoW}| market for quicklime, in pieces, loose | Cut-off, U	0.055	kg
Refractory, basic, packed {GLO}| market for | Cut-off, U	0.0135	kg
Pre-reduced V_2O_5 magnetite adapted from Iron pellet {RoW}| production | Cut-off, U	1.46	kg
Vanadium pentaoxide V_2O_5 bearing cast iron Fe	1.32	kg
Aluminium, wrought alloy {GLO}| market for | Cut-off, U	0.000015	kg
Argon, liquid {RoW}| market for argon, liquid | Cut-off, U	0.00329	kg
Cast iron {GLO}| market for | Cut-off, U	0.000052	kg
Diesel, burned in building machine {GLO}| market for | Cut-off, U	0.00346	MJ
Ethylene glycol {GLO}| market for | Cut-off, U	3.3E-08	kg
Ferrochromium, high-carbon, 68% Cr {GLO}| market for | Cut-off, U	0.00011	kg
Ferromanganese, high-coal, 74.5% Mn {GLO}| market for | Cut-off, U	0.000045	kg
Ferrosilicon {GLO}| market for | Cut-off, U	0.0037	kg
Molybdenum trioxide {GLO}| market for | Cut-off, U	0.000014	kg
nickel, class 1 {GLO}| market for nickel, class 1 | Cut-off, U	0.000033	kg
Propane, burned in building machine {GLO}| market for | Cut-off, U	0.00273	MJ
Electricity, medium voltage {CN}| market group for | Cut-off, U	0.54	kWh
Electricity, low voltage {CN}| market group for | Cut-off, U	0.0346	kWh
Heat, district or industrial, natural gas {RoW}| market for heat, district or industrial, natural gas | Cut-off, U	1.23	MJ
**Outputs**		
Vanadium Slag (25% V_2O_5)	0.0613	kg
Steel, low alloyed	1.2	kg
Benzene to air	2.31E-06	kg

Cadmium to air	3.65E-08	kg
Carbon monoxide, fossil to air	0.00232	kg
Chromium to air	1.25E-06	kg
Copper to air	2.31E-07	kg
Dioxins (TEQ) to air	4.54E-12	kg
Hydrocarbons, aromatic to air	7.7E-05	kg
Hydrogen chloride to air	5.2E-06	kg
Hydrogen fluoride to air	2.35E-06	kg
Lead to air	1.81E-06	kg
Mercury to air	2.22E-06	kg
Nickel to air	7.01E-07	kg
Nitrogen oxides to air	0.00018	kg
PAH, polycyclic aromatic hydrocarbons to air	3.73E-08	kg
Particulates, < 2.5 um to air	0.000166	kg
Particulates, > 10 um to air	5.86E-05	kg
Particulates, > 2.5 um, and < 10um to air	1.6596	kg
Polybrominated biphenyls to air	2.33E-08	kg
Sulfur dioxide to air	0.000077	kg
Water to air	0.002021	kg
Zinc to air	2.29E-05	kg
Argon-40/kg to air	0.00329	kg
Benzene, hexachloro- to air	2E-08	kg
Water to water	0.003195	kg
Chloride to water	1.8E-06	kg
Chromium VI to water	1.9E-09	kg
Inert waste, for final disposal {RoW}| market for inert waste, for final disposal | Cut-off, U	0.005	kg
electric arc furnace dust {RoW}| market for electric arc furnace dust | Cut-off, U	0.0096	kg
electric arc furnace slag {RoW}| market for electric arc furnace slag | Cut-off, U	0.0498	kg
Scrap steel {RoW}| market for scrap steel | Cut-off, U	0.000052	kg
Spent solvent mixture {Europe without Switzerland}| market for spent solvent mixture | Cut-off, U	3.3E-08	kg
Blast furnace slag {GLO}| market for | Cut-off, U	0.0928	kg

Table S4: Life cycle inventory for Vanadium Pentaoxide V_2O_5 (adapted from Da Silva et al 2021 [35]).

**Dataset**	**Amount**	**Unit**
**Inputs**		
Vanadium Slag (25% V_2O_5)	1.35	kg
ammonium sulfate {RoW}| market for ammonium sulfate | Cut-off, U	0.31	kg
Soda ash, dense {GLO}| market for | Cut-off, U	0.37	kg
Sodium sulfate, anhydrite {RoW}| market for | Cut-off, U	0.5	kg

Sulfuric acid {RoW}| market for sulfuric acid | Cut-off, U	0.46	kg
Transport, freight train {CN}| market for | Cut-off, U	0.46	tkm
Transport, freight, lorry 16-32 metric ton, EURO6 {RER}| transport, freight, lorry 16-32 metric ton, EURO6 | Cut-off, U	0.0771	tkm
water, deionised {RoW}| market for water, deionised | Cut-off, U	3.16	kg
Electricity, medium voltage {CN}| market group for | Cut-off, U	0.2	kWh
Heat, district or industrial, natural gas {RoW}| market for heat, district or industrial, natural gas | Cut-off, U	0.93	MJ
**Outputs**		
Vanadium Pentaoxide V_2O_5	1.00	kg
Sodium sulfate, anhydrite	1.00	kg
Carbon dioxide to air	0.16	kg
Oxygen to air	0.0564	kg
Sulfur dioxide to air	0.23	kg
Water to air	0.0845	kg
Scrap steel {RoW}| market for scrap steel | Cut-off, U	0.065	kg
Spent solvent mixture {Europe without Switzerland}| market for spent solvent mixture | Cut-off, U	0.0632	kg
Slag from metallurgical grade silicon production {GLO}| market for | Cut-off, U	0.47	kg

Table S5: Life cycle inventory for Vanadium Cloride VCl_3 (adapted from Da Silva et al 2021 [35]).

Dataset	Amount	Unit
**Inputs**		
Vanadium Pentaoxide V_2O_5	0.57813	kg
Hydrochloric acid, without water, in 30% solution state {RoW}| market for | Cut-off, U	0.69536	kg
Hydrogen, liquid {RoW}| market for | Cut-off, U	0.00641	kg
Electricity, medium voltage {CN}| market group for | Cut-off, U	0.33	kWh
**Outputs**		
Vanadium Cloride VCl_3	1.0000	kg
Water to air	0.2632	kg

Table S6: Life cycle inventory for Vanadium (adapted from Titanium primary, triple-melt {GLO}\| titanium production, primary, triple melt \| Cut-off, U).

Dataset	Amount	Unit
**Inputs**		
Vanadium Cloride VCl_3	3.087	kg
Argon, liquid {RER}| market for argon, liquid | Cut-off, U	0.030972	kg
Argon, liquid {RoW}| market for argon, liquid | Cut-off, U	0.147028	kg
Magnesium {GLO}| market for | Cut-off, U	0.01145	kg
Electricity, high voltage {GLO}| market group for | Cut-off, U	27.84	kWh

Heat, district or industrial, natural gas {GLO}| market group for | Cut-off, U	9	MJ
**Outputs**		
Vanadium (adapted from Titanium primary, triple-melt {GLO}| titanium production, primary, triple melt | Cut-off, U)	1.000	kg

Table S7: Life cycle inventory for Zirconium tetrachloride (adapted from Titanium tetrachloride {GLO}| production | Cut-off, U).

Dataset	Amount	Unit
**Inputs**		
Zirconium oxide {GLO}| market for | Cut-off, U	1.192	kg
Chlorine, liquid {RER}| market for chlorine, liquid | Cut-off, U	0.030972	kg
Chlorine, liquid {RoW}| market for chlorine, liquid | Cut-off, U	0.147028	kg
Coke {GLO}| market for | Cut-off, U	0.016	kg
Electricity, high voltage {GLO}| market group for | Cut-off, U	014	kWh
Heat, district or industrial, natural gas {GLO}| market group for | Cut-off, U	2.1	MJ
**Outputs**		
Zirconium (adapted from Titanium primary, triple-melt {GLO}| titanium production, primary, triple melt | Cut-off, U)	1.000	kg

Table S8: Life cycle inventory for Zirconium (adapted from Titanium primary, triple-melt {GLO}| titanium production, primary, triple melt | Cut-off, U).

Dataset	Amount	Unit
**Inputs**		
Zirconium tetrachloride (adapted from Titanium tetrachloride {GLO}| production | Cut-off, U)	4.000	kg
Argon, liquid {RER}| market for argon, liquid | Cut-off, U	0.030972	kg
Argon, liquid {RoW}| market for argon, liquid | Cut-off, U	0.147028	kg
Magnesium {GLO}| market for | Cut-off, U	0.016	kg
Electricity, high voltage {GLO}| market group for | Cut-off, U	27.84	kWh
Heat, district or industrial, natural gas {GLO}| market group for | Cut-off, U	9	MJ
**Outputs**		
Zirconium (adapted from Titanium primary, triple-melt {GLO}| titanium production, primary, triple melt | Cut-off, U)	1.000	kg

Table S9: Life cycle inventory for $Pd_{77}Ag_{23}$ membrane.

Dataset	Amount	Unit
**Inputs**		
Palladium {GLO}| market for | Cut-off, U	0.77	kg

Silver {GLO}| market for | Cut-off, U	0.23	kg
**Outputs**		
$Pd_{77}Ag_{23}$ membrane	1.00	kg

Table S10: Life cycle inventory for $V_{93}Pd_7$ membrane with Pd coatings.

**Dataset**	**Amount**	**Unit**
**Inputs**		
Vanadium (adapted from Titanium primary, triple-melt {GLO}| titanium production, primary, triple melt | Cut-off, U)	0.86	kg
Palladium {GLO}| market for | Cut-off, U	0.37	kg
**Outputs**		
$V_{93}Pd_7$ membrane with Pd coatings	1.00	kg

Table S11: Life cycle inventory for $Ti_{30}Zr_9V_{34}Pd_{27}$ membrane (ZrVTiPd2) with Pd coatings.

**Dataset**	**Amount**	**Unit**
**Inputs**		
Titanium primary, triple-melt {GLO}| market for | Cut-off, U	0.21	kg
Zirconium adapted from Titanium primary, triple-melt {GLO}| titanium production, primary, triple melt | Cut-off, U	0.12	kg
Palladium {GLO}| market for | Cut-off, U	0.59	kg
Vanadium (adapted from Titanium primary, triple-melt {GLO}| titanium production, primary, triple melt | Cut-off, U)	0.25	kg
**Outputs**		
$Ti_{30}Zr_9V_{34}Pd_{27}$ membrane ZrVTiPd2	1.00	kg

Table S12: Life cycle inventory for $Ti_{13}Zr_{52}V_{12}Pd_{23}$ membrane (ZrVTiPd3) with Pd coatings.

**Dataset**	**Amount**	**Unit**
**Inputs**		
Titanium primary, triple-melt {GLO}| market for | Cut-off, U	0.07	kg
Zirconium adapted from Titanium primary, triple-melt {GLO}| titanium production, primary, triple melt | Cut-off, U	0.56	kg
Palladium {GLO}| market for | Cut-off, U	0.44	kg
Vanadium (adapted from Titanium primary, triple-melt {GLO}| titanium production, primary, triple melt | Cut-off, U)	0.07	kg
**Outputs**		
$Ti_{13}Zr_{52}V_{12}Pd_{23}$ membrane (ZrVTiPd3)	1.00	kg

Table S13: Life cycle inventory for $Ti_{19}Zr_{39}V_{20}Pd_{22}$ membrane (ZrVTiPd4) with Pd coatings.

Dataset	Amount	Unit
**Inputs**		
Titanium primary, triple-melt {GLO}| market for | Cut-off, U	0.12	kg
Zirconium adapted from Titanium primary, triple-melt {GLO}| titanium production, primary, triple melt | Cut-off, U	0.45	kg
Palladium {GLO}| market for | Cut-off, U	0.69	kg
Vanadium (adapted from Titanium primary, triple-melt {GLO}| titanium production, primary, triple melt | Cut-off, U)	0.13	kg
**Outputs**		
$Ti_{19}Zr_{39}V_{20}Pd_{22}$ membrane (ZrVTiPd4)	1.00	kg

Table S14: Life cycle inventory for $Ti_{23}Zr_{11}V_{27}Pd_{39}$ membrane (ZrVTiPd6) with Pd coatings.

Dataset	Amount	Unit
**Inputs**		
Titanium primary, triple-melt {GLO}| market for | Cut-off, U	0.14	kg
Zirconium adapted from Titanium primary, triple-melt {GLO}| titanium production, primary, triple melt | Cut-off, U	0.13	kg
Palladium {GLO}| market for | Cut-off, U	0.74	kg
Vanadium (adapted from Titanium primary, triple-melt {GLO}| titanium production, primary, triple melt | Cut-off, U)	0.18	kg
**Outputs**		
$Ti_{23}Zr_{11}V_{27}Pd_{39}$ membrane (ZrVTiPd6)	1.00	kg
